# Supplementary material for: Gene Signatures Associated with Temporal Rhythm as Diagnostic Markers of Major Depressive Disorder and Their Role in Immune Infiltration
Source: Int J Mol Sci. 2022 Sep 30;23(19):11558. doi: 10.3390/ijms231911558 (PMC9570069; doi:10.3390/ijms231911558)
Supplement: Supplementary file 1 [file ijms-23-11558-s001.zip › ijms-1925675-supplementary.pdf]

Supplementary Table S1 GSEA analysis

ID	Description	setSize	enrichmentScore	NES	pvalue	rank	leading_edge	core_enrichment
KEGG_ALLOGRAFT_REJECTION	KEGG_ALLOGRAFT_REJECTION	2	0.98941	1.731723	0.000201	9	tags=100%, list=1%, signal=99%	HLA-DRA/HLA-DPB1
KEGG_ASTHMA	KEGG_ASTHMA	2	0.98941	1.731723	0.000201	9	tags=100%, list=1%, signal=99%	HLA-DRA/HLA-DPB1
WP_SPINAL_CORD_INJURY	WP_SPINAL_CORD_INJURY	12	-0.58409	-2.09856	0.000926	109	tags=67%, list=16%, signal=57%	PTGS2/GADD45A/FOS/GRIN1/CCL2/EGR1/ZFP36/PDYN
REACTOME_NEUTROPHIL_DEGRANULATION	REACTOME_NEUTROPHIL_DEGRANULATION	20	0.467819	2.15711	0.001118	99	tags=50%, list=15%, signal=44%	DSP/HSPA6/CTSH/C3/PTPRC/TTR/PGM2/TYROBP/CYBA/GLIPR1
PID_AP1_PATHWAY	PID_AP1_PATHWAY	8	-0.70169	-2.14712	0.001134	97	tags=75%, list=15%, signal=65%	FOS/CCL2/DUSP1/EGR1/PENK/FOSL2
WP_PANCREATIC_ADENOCARCINOMA_PATHWAY	WP_PANCREATIC_ADENOCARCINOMA_PATHWAY	5	-0.79635	-2.00875	0.001555	140	tags=100%, list=21%, signal=79%	PIK3CD/PLD1/GADD45A/VEGFA
